# Enabling scalable ferroelectric-based future generation vertical NAND flash with bonding-friendly architecture: strategies for erase and disturb optimization

**DOI:** 10.1039/d5na00844a

**Published:** 2025-12-24

**Authors:** Ickhyun Song, Juhyun Kim, Seungmin Lee, Ilho Myeong

**Affiliations:** a Department of Electronic Engineering, Hanyang University Seoul 04763 Republic of Korea; b Inter-University Semiconductor Research Center, School of Electrical Engineering and Computer Science, Seoul National University Seoul 08826 South Korea; c Semiconductor R&D Center, Samsung Electronics Co. Ltd South Korea; d Department of Electronics Engineering, Myongji University Yongin-Si 17058 Korea myoung2212@mju.ac.kr

## Abstract

We propose a novel ferroelectric VNAND (Fe-VNAND) architecture based on a TCAT (Terabit Cell Array Transistor) structure, integrating an amorphous IGZO channel and a band-engineered filler insulator for enhanced erase and disturbance characteristics. To overcome the limitations of poor hole transport in IGZO, a tailored erase (ERS) scheme employing stepped dummy word-line biasing is introduced, which effectively mitigates over-erasure at the bottom of the NAND string and enables reliable bitline sensing. By optimizing the doping overlap of the source line (*L*_OV_) and operating the select word-line at low voltage (3 V), we demonstrate significantly reduced read disturbance and improved threshold voltage uniformity. Furthermore, the application of a band-engineered oxide/nitride filler structure enhances hole injection during ERS, leading to a 30% increase in memory window and a two-order-of-magnitude improvement in erase speed. Our findings suggest that the proposed structure and scheme are highly compatible with existing TCAT flows and scalable to future high-density ferroelectric memory systems. These innovations pave the way for energy-efficient, disturbance-tolerant 3D Fe-VNAND applicable to AI accelerators and edge computing platforms.

## Abstract 2

We propose a novel ferroelectric VNAND (Fe-VNAND) architecture based on a TCAT (Terabit Cell Array Transistor) structure, integrating an amorphous IGZO channel and a band-engineered filler insulator for enhanced erase and disturbance characteristics.

## Graphical abstract

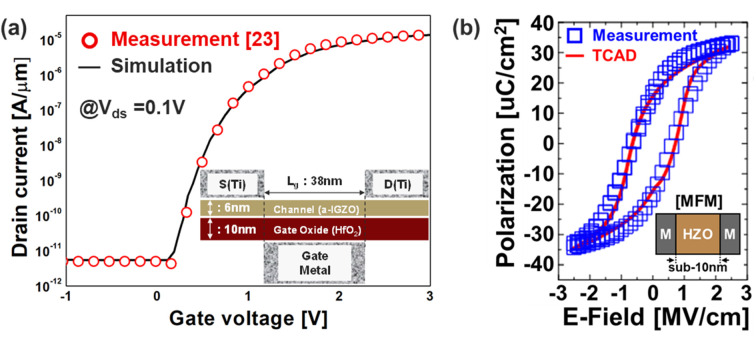

## Introduction

Since the emergence of the vertical dimension in NAND Flash technology, significant efforts have been made to achieve higher memory capacity within the same chip area by extending cells in the vertical (*z*-axis) direction.^1,15^ Vertical NAND (V-NAND) architectures have become essential for meeting the growing demand for high-density non-volatile memory in applications such as data center, mobile devices, and AI accelerators. A key technology used in conventional V-NAND is the charge trap nitride (CTN) structure,^24^ which stores charges in a nitride layer to represent data. However, as the industry continues to push toward more aggressive scaling, particularly in the vertical pitch (*z*-pitch) and horizontal spacing, the conventional charge trap nitride (CTN)-based V-NAND architectures face critical challenges. These include not only the high program/erase voltages but also the increasing susceptibility to reliability degradation mechanisms such as trap-assisted tunneling (TAT) and time-dependent dielectric breakdown (TDDB). Moreover, as stack height increases beyond 200 layers, the cumulative voltage drop and word-line interference become non-negligible, making the low-voltage operation of emerging devices like Fe-VNAND even more attractive.^7^ These high voltages lead to reliability issues, increased power consumption, and limitations in further shrinking the cell dimensions.

To address these challenges, ferroelectric-based V-NAND (Fe-VNAND) has recently attracted attention as a promising alternative.^20^ Ferroelectric materials possess remanent polarization characteristics, enabling data storage at much lower voltages compared to CTN counterparts. This makes them particularly well-suited for low-power applications and offers considerable advantages in terms of operation voltage, programming speed, and cell scalability.^2–4^ In particular, Fe-VNAND shows strong potential in enabling tighter *z*-pitch scaling, which is essential for increasing storage density without enlarging the die size.

Recent studies have primarily focused on Fe-VNAND devices that use polycrystalline silicon (poly-Si) as the channel material, owing to its compatibility with existing semiconductor processes.^5–8^ While poly-Si offers certain benefits, such as acceptable mobility and well-established fabrication techniques, it suffers from significant drawbacks at the interface with the gate oxide. The poly-Si/gate oxide interface tends to have high defect densities and poor interface quality, which degrade the subthreshold slope (SS), reduce mobility, and lead to increased charge trapping phenomena.^9^ These effects ultimately limit the performance and reliability of the memory device, particularly as scaling continues.

In order to overcome these issues, researchers have explored the use of oxide semiconductors, such as indium–gallium–zinc–oxide (IGZO),^21–23^ in Fe-VNAND structures. IGZO offers excellent material properties including high electron mobility, low leakage current, and a smooth interface with the ferroelectric layer. Importantly, it eliminates the need for an additional insulating buffer between the channel and the ferroelectric, which can otherwise degrade device characteristics. As a result, IGZO-based Fe-VNAND structures demonstrate improved memory properties, such as enhanced endurance, longer retention time, and a larger memory window (M. W.).^10^ However, a critical limitation of IGZO lies in its poor hole transport characteristics, which makes it difficult to implement conventional erase (ERS) operations that rely on efficient hole injection.

To mitigate this problem, a novel approach involving a P-type silicon filler inserted into the center of the vertical oxide filler was proposed.^11^ This design helps facilitate hole injection during the erase process by providing a conductive path for hole movement, thereby enabling ERS functionality in IGZO channel-based Fe-VNAND devices. Building upon this prior work, our study adopts the Terabit Cell Array Transistor (TCAT) structure, which includes a P-type silicon filler at the center of the vertical stack. In addition to replacing the conventional CTN with a ferroelectric layer, we further optimize the device architecture and operating conditions to achieve enhanced performance.

In this paper, we also employed Terabit Cell Array Transistor (TCAT) structure with P-type silicon filler in the center, also including ferroelectric layer rather than charge-trap-nitride (CTN). We optimize device specification and operating voltage, and propose a new ERS scheme. We also apply band-engineered (BE) filler insulator to the device to improve erase performance. In addition, read disturbance characteristics are compared within the available read voltage (*V*_read_) range, offering insights into its potential for next-generation high-density memory applications.

### Simulation setup

**Fig. 1:**
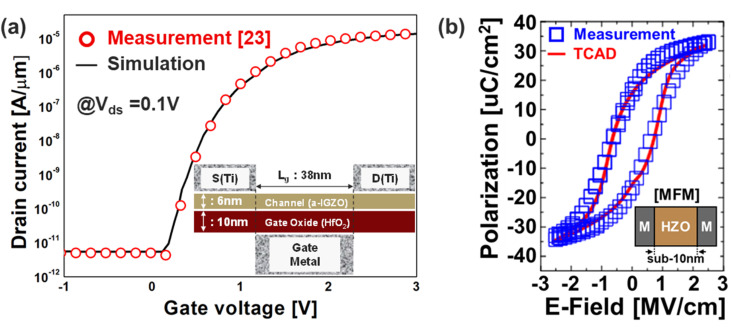
(a) TCAD calibration of *I*–*V* characteristics with a-IGZO MOSFET measurement data, and (b) TCAD calibration of polarization characteristics with MFM structure measurement data.

**Fig. 2:**
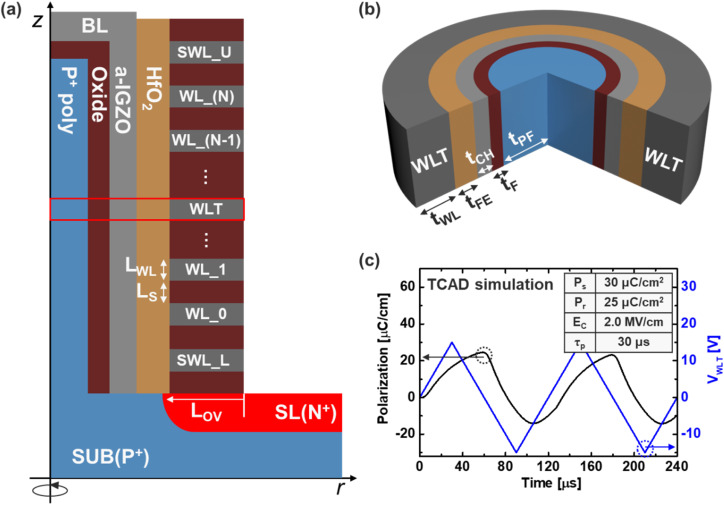
Schematic of the (a) Fe-VNAND and (b) unit cell. (c) Pr. and *V*_WLT_ in the simulation as a function of time at a ramp time of 30 µs.

In order to investigate the characteristics of a-IZGO channel-based Fe-VAND, we first conduct TCAD calibration steps. *I*_D_–*V*_G_ data of a-IGZO MOSFET is employed to calibrate the TCAD simulation. Here, in order to accurately reproduce the electrical behavior of the Fe-VNAND structure with an amorphous IGZO channel,^17,18^ we adopted material parameters derived from the comprehensive numerical study reported by Fung *et al.*^12^ This model has been widely validated for a-IGZO thin-film transistors (TFTs) and captures key aspects of its electronic structure, including the presence of localized tail states and oxygen-vacancy-induced subgap states. Notably, the effective density of states in the conduction and valence bands was set to 5 × 10^18^ cm^−3^, and the energy bandgap of a-IGZO was defined as 3.05 eV, with an electron affinity of 4.16 eV. The presence of exponential tail states was modeled with densities of 1.55 × 10^20^ cm^−3^ eV^−1^ at both band edges, and characteristic slopes of *E*_a_ = 13 meV and *E*_d_ = 120 meV for the conduction and valence bands, respectively. The tail states near the conduction band were modeled as acceptor-like traps, whereas those near the valence band were modeled as donor-like traps. Additionally, oxygen-vacancy (OV) states, which are known to significantly influence charge trapping and switching behavior, were incorporated using a Gaussian distribution with a peak density of 6.5 × 10^16^ cm^−3^ eV^−1^, a mean energy (*λ*) of 2.9 eV, and a standard deviation (*σ*) of 0.1 eV. In order to reflect their properties, these OV states—despite being located near the conduction band—were modeled as donor-like traps in the simulation. The carrier mobilities were set to *µ*_n_ = 15 cm^2^ V^−1^ for electrons and *µ*_p_ = 0.1 cm^2^ V^−1^ for holes, consistent with experimentally extracted data for sputtered a-IGZO films. All IGZO parameters follow ref. 12, with only minor (<10%) adjustments made to mobility and tail-state slope for accurate subthreshold matching. Accordingly, excellent agreement was observed between TCAD and measurements as shown in Fig. 1(a), indicating a-IGZO characteristics were successfully implemented. By incorporating this parameter set, the model effectively captures the inherent asymmetry in electron and hole transport in IGZO, which is critical for accurately analyzing polarization switching and erase performance in Fe-VNAND devices. Furthermore, the simulated characteristics of the IGZO channel, including subthreshold behavior and leakage suppression, align well with experimental trends reported in previous oxide TFT literature. On the other hand, TCAD calibration of polarization characteristics is then performed. In order to reproduce the polarization (Pr.) characteristics of the Hf_0.5_Zr_0.5_O_2_ (HZO), Preisach model is introduced to the material with dielectric constant of 25. The simulated Pr., calibrated using the fabricated MFM capacitor in Fig. 2(c), is based on *P*_s_ (30 µC cm^−2^), *P*_r_ (25 µC cm^−2^), *E*_c_ (1.5 MV cm^−1^), *τ*_p_ (250 ns). As a result, good agreement between TCAD and measurements in Fig. 1(b) shows that Pr. characteristics of HZO were successfully implemented.

Fig. 2(a) shows a schematic cross-section of the Fe-VNAND structure used in TCAD: a terabit cell array transistor (TCAT) structure. Here, *L*_OV_ stands for the overlap length between doping region and memory hole. Fe-VNAND string consists of upper and lower select word-line (SWL_U/L), and seven word-lines (WLs). Table 1 provides the device specification. Fig. 2(b) is a 3D image of the unit cell structure, which is part of (a). Since the structure under investigation includes IGZO channel on one side, which is expected to exhibit relatively slower switching dynamics, *τ*_p_ was adjusted to be an order of magnitude higher than the calibrated value.^9,13^ Accordingly, Fig. 2(d) presents the simulated Pr. when *V*_WLT_ was applied to (b), with *τ*_p_ increased to 30 µs while keeping the other parameters fixed. Due to the poor hole characteristic of IGZO, relatively less Pr. occurs at negative *V*_WLT_.

**Table 1:** Device parameters used in the simulation

Parameters	Value	Parameters	Value
P-filler radius (*t*_PF_)	22 nm	WL thickness (*t*_WL_)	40 nm
Filler insulator thickness (*t*_F_)	8 nm	WL length (*L*_WL_)	22 nm
Channel thickness (*t*_CH_)	8 nm	Spacer length (*L*_SP_)	22 nm
Ferroelectric layer thickness (*t*_FE_)	10 nm	Blocking oxide thickness (*t*_BOX_)	6.5 nm

**Fig. 3:**
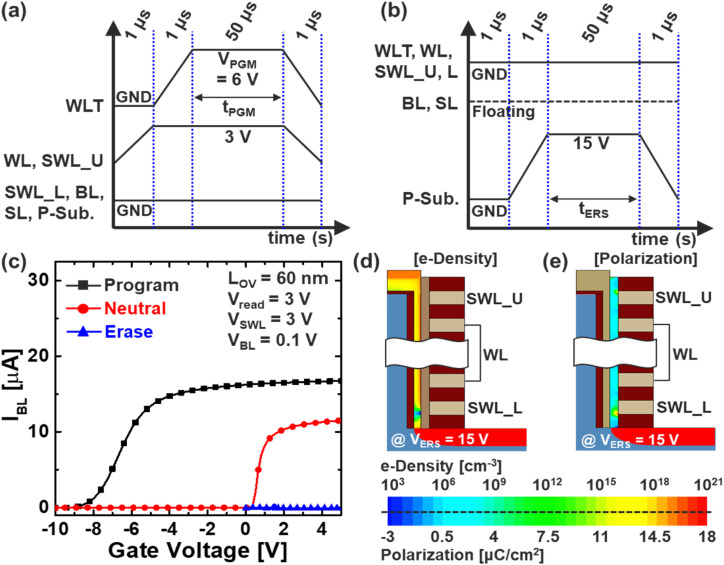
Bias scheme of (a) program and (b) erase operation, and (c) *I*–*V* transfer curves of Fe-VNAND in programmed, neutral and erased state. Simulated (d) electron density of a-IGZO channel and (e) Pr. of FE layer after erase operation.

Fig. 3(a) and (b) show the conventional bias schemes for program (PGM) and erase operations in IGZO-channel based VNAND structures, respectively.^11^ During erase, the bit line (BL) may serve as a path for hole extraction, so it is kept floating to prevent unwanted current flow. Fig. 3(c) illustrates the *I*–*V* transfer curves for programmed, neutral, and erased states. However, in the erased state, *I*_BL_–*V*_WLT_ characteristics are not clearly observed due to insufficient channel conduction, which suggests an abnormal erase condition near the string bottom. As shown in Fig. 3(d), the channel under SWL_L becomes depleted, resulting in low electron density and suppressed current flow. This depletion is caused by polarization (Pr.) in the ferroelectric layer beneath SWL_L, as shown in Fig. 3(e), which is induced by the 15 V bias applied to both the substrate and P^+^ filler. This creates a strong electric field at the lower WL corner, leading to excessive polarization accumulation and significantly increasing the local threshold voltage (*V*_th_), such that normal read operation becomes unfeasible for the over-erased cell. Consequently, the erased cell becomes electrically isolated, impairing sensing reliability and degrading overall string performance. Furthermore, this phenomenon can result in inconsistent charge distribution across the vertical channel, potentially affecting adjacent cells through parasitic coupling and increasing read variation.

### Bias scheme of erase operation

**Fig. 4:**
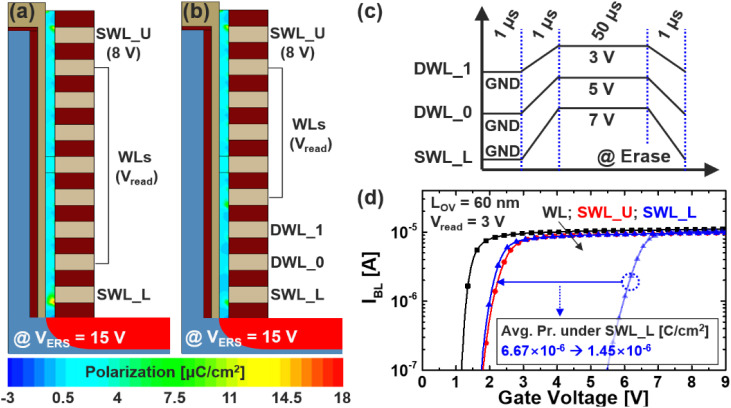
Simulated Pr. after (a) conventional erase and (b) proposed scheme. (c) Proposed ERS scheme, and (d) *I*–*V* transfer curves for WL, SWL_U; L.

In order to reduce the over-erase issue observed at the lower select word-line (SWL_L), we introduce a novel erase bias scheme that utilizes two additional WLs at the bottom of the string, designated as dummy WLs (DWLs). Unlike conventional schemes where SWL_L is solely biased and the surrounding WLs are grounded, the proposed method applies stepped voltages of 7 V, 5 V, and 3 V to SWL_L, DWL_0, and DWL_1, respectively, as illustrated in Fig. 4(c). This voltage gradient across the bottom word-lines is designed to spatially modulate the vertical electric field, thereby reducing abrupt field concentrations near the lower WL corner and preventing excessive polarization. If a high voltage is applied only to SWL_L, a strong vertical field is also induced in neighboring cells above, which results in unwanted Pr. formation and residual disturbance in adjacent memory layers. Although this may reduce the degree of polarization compared to the conventional full-bias case, it still does not sufficiently suppress the threshold voltage shift to enable reliable read operation. By employing a distributed bias approach through the use of two dummies, the electric field at the lower end of the stack is more gradually tapered, leading to a significant reduction in over-erased behavior. This improvement is clearly demonstrated in Fig. 4(a) and (b), which show the spatial distribution of simulated Pr. after applying the conventional and proposed erase schemes, respectively. In Fig. 4(b), the excessive polarization that previously formed at the lower corner of SWL_L is markedly diminished, leading to a more uniform polarization profile across the string. This effect is further corroborated in Fig. 4(d), where the threshold voltage (*V*_th_) of SWL_L, indicated by the blue curve with triangle markers, is reduced to a level comparable to that of other word-lines. Additionally, the average polarization density beneath SWL_L decreases significantly from 6.67 to 1.45 µC cm^−2^, demonstrating the efficacy of the proposed scheme in suppressing localized over-erasure while maintaining array-level consistency.

**Fig. 5:**
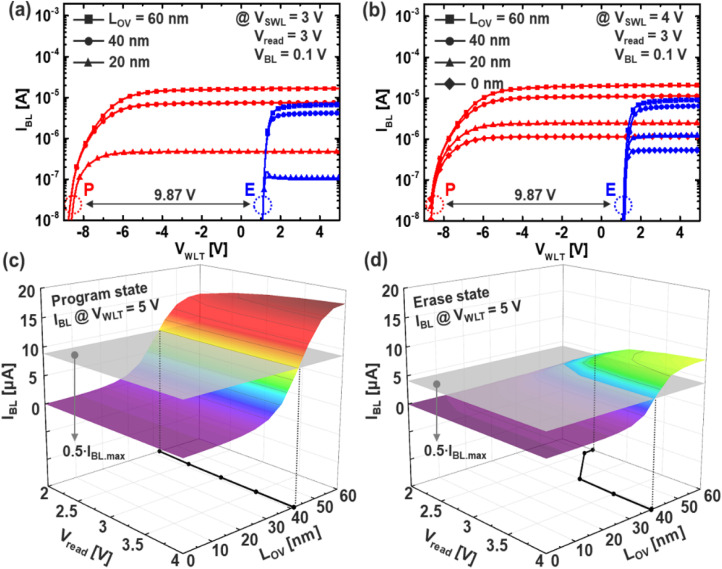
Simulated *I*_BL_ (a) for various *L*_OV_ and (b) *V*_read_ as a function of *V*_WLT_. 3D surface plot of *I*_BL_ for *L*_OV_ and *V*_read_ after (c) program and (d) erase operation.

By introducing the devised erase (ERS) scheme based on multi-level dummy word-line biasing, it becomes possible to stably operate the memory array at a lower select word-line voltage, specifically *V*_SWL_ = 3 V. This low-voltage operation is critical for minimizing stress on peripheral circuits and improving the energy efficiency of the erase process. Fig. 5(a) and (b) present the *I*–*V* transfer characteristics in both programmed and erased states as a function of varying source-line overlap length (*L*_OV_), under *V*_SWL_ = 3 V and 4 V, respectively. Here, *L*_OV_ refers to the physical length over which the n^+^-doped source line overlaps with the vertical channel string, as illustrated in Fig. 2(a).

As shown in the figures, when V_SWL is low, the ability to induce inversion beneath SWL_L becomes strongly dependent on L_OV. A smaller L_OV reduces the erased-state string current (*I*_BL_) by limiting channel formation in the lower segment, underscoring the need for proper L_OV selection to maintain uniform drivability across WLs. In this work, L_OV = 60 nm was chosen as a balanced design point between erase performance and V_th uniformity, and read operation was evaluated at V_SWL = 3 V to minimize energy consumption and disturbance.

Fig. 5(c) and 4(d) show the 3D surface plots of *I*_BL_ with respect to both *V*_read_ and *L*_OV_ in the programmed and erased states, respectively. The gray cross-sectional plane denotes 50% of the maximum current, serving as a practical threshold to assess readability. From the intersection of the surface and the gray plane, it is observed that in the programmed state, *I*_BL_ remains relatively insensitive to *L*_OV_ across all *V*_read_ values, suggesting robust conduction. In contrast, in the erased state, *I*_BL_ decreases drastically as *L*_OV_ is reduced—particularly when *V*_read_ < 3 V—emphasizing the sensitivity of erased-state conduction to structural overlap and read bias.

Because smaller *V*_read_ is desirable from a read disturbance mitigation perspective, we fixed *V*_read_ = 3 V as a trade-off point that ensures acceptable *I*_BL_ while reducing unnecessary stress on the ferroelectric layer, thereby preserving polarization stability during repeated reads.

**Fig. 6:**
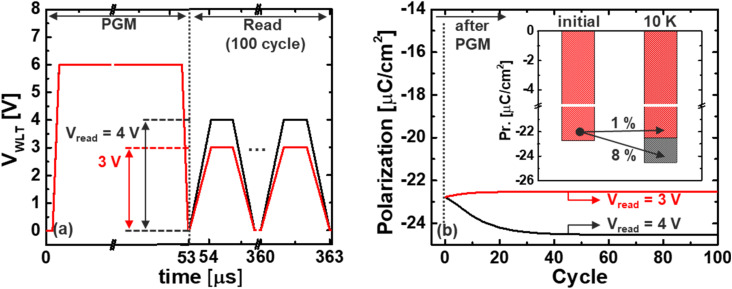
(a) Pulse of *V*_WLT_ including program and read operation, and (b) the change in Pr. over read cycles at *V*_read_ = 3 and 4 V. The inset further includes read-disturb results up to 10^4^ cycles, validating stable long-term Pr. retention under low *V*_read_ operation.

To further investigate the impact of repeated read operations on device stability and data integrity, a dedicated read disturbance analysis was conducted under the proposed low-voltage operation scheme. Fig. 6(a) illustrates the pulse scheme used in this study, in which 100 consecutive read pulses were applied to the target word-line (WL) following a standard program (PGM) operation. Between each pulse, the remanent polarization (Pr.) of the ferroelectric layer was extracted to assess any cumulative degradation or fatigue effects. This methodology enables a direct evaluation of the influence of read cycling on ferroelectric switching reliability, which is particularly critical in IGZO-based Fe-VNAND architectures known to exhibit asymmetric carrier transport and limited hole mobility.

As shown in Fig. 6(b), the extracted Pr. values reveal a striking difference in retention stability depending on the applied read voltage. When *V*_read_ = 3 V, the cumulative change in Pr. over 100 read cycles is significantly reduced, demonstrating approximately 8× lower degradation compared to scenarios involving higher read voltages such as 4 V. This substantial suppression of polarization fatigue at lower *V*_read_ can be attributed to the reduced electric field stress applied to the ferroelectric layer during each read access, which in turn minimizes undesired dipole reorientation and charge injection events.

These findings reinforce the strategic value of operating at a minimized *V*_read_, not only from the standpoint of energy efficiency, but also in terms of long-term memory reliability. In practical applications, such voltage optimization can substantially extend device endurance and reduce the risk of read-disturb-induced failures, which are key concerns in scaled 3D memory stacks and AI-inference workloads involving frequent read accesses.

## Band engineering and read disturbance

**Fig. 7:**
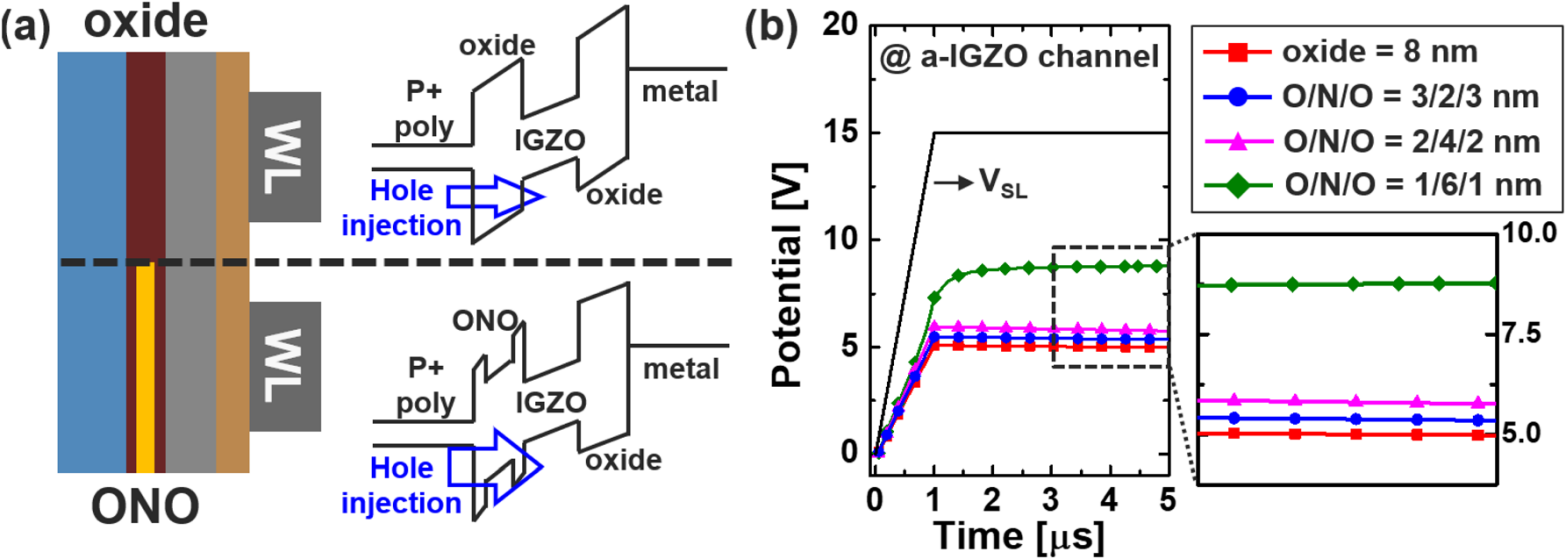
(a) Band of oxide and BE-oxide filler insulator, and (b) channel potential for various condition of filler insulator.

**Fig. 8:**
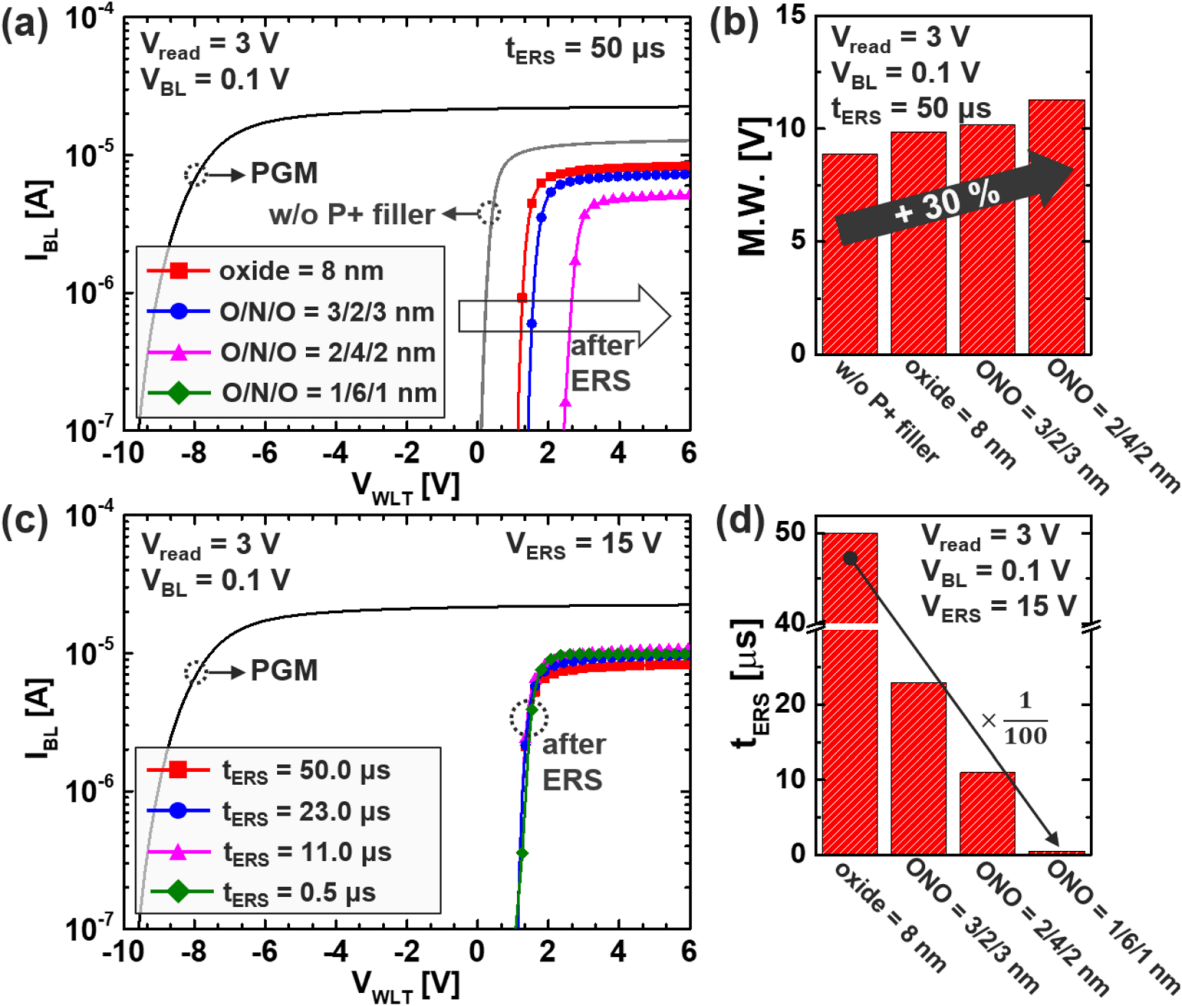
(a) *I*_BL_ and (b) M. W. under the same *t*_ERS_ for various filler structure, and (c) *I*_BL_ and (d) *t*_ERS_ when targeting the same *V*_th_ for various filler structure.

In the baseline configuration of the Fe-VNAND device, silicon dioxide (SiO_2_) was employed as the filler insulator material between the ferroelectric layer and the P^+^-doped vertical silicon pillar. This configuration offers fabrication simplicity and good chemical stability; however, it inherently limits the hole injection efficiency during the erase (ERS) operation.^14^ This limitation arises from the relatively high energy barrier at the interface between the oxide filler and the IGZO channel, which suppresses hole tunneling from the P^+^ filler into the channel. Since effective hole injection is essential for generating an internal electric field that can reverse the polarization state of the ferroelectric (FE) layer during erase, the SiO_2_-only structure leads to incomplete or inefficient switching, thereby constraining both memory window and ERS speed. To address this limitation, as illustrated in Fig. 7(a), a band-engineered (BE) filler design was introduced. In this modified structure, a nitride (N) region is incorporated into the oxide filler stack to form a composite insulator. The purpose of this structural engineering is to lower the effective tunneling barrier height for holes by improving the energy band alignment between the P^+^ filler and the IGZO channel. The conduction and valence band offsets between IGZO and SiN_*x*_ are smaller than those for SiO_2_, thereby facilitating hole transport across the interface.^13,16,19^ This band engineering approach creates a smoother potential landscape, promoting more efficient carrier injection and enabling more uniform polarization reversal throughout the ferroelectric layer. The effect of this structural modification is clearly reflected in the simulated channel potential profiles under various filler compositions, as shown in Fig. 7(b). As the nitride proportion within the filler increases, the effective tunneling barrier is lowered, enabling faster hole transport from the p^+^ silicon filler into the channel and promoting stronger hole accumulation. This tunneling-driven potential rise establishes more favorable conditions for polarization switching and ensures that the switching proceeds uniformly along the vertical direction, which is essential for tall VNAND stacks. From a device reliability perspective, uniform switching helps suppress localized over- or under-programming, thereby improving overall bitline sensing consistency. As a result of the improved hole injection enabled by the BE filler, both the memory window (M. W.) and the ERS performance exhibit substantial enhancement. Compared to the baseline SiO_2_-only configuration, the incorporation of even a thin nitride interlayer leads to a measurable increase in polarization change, and in turn, threshold voltage modulation. In later sections (see Fig. 8), we show that the memory window increases by approximately 30%, and the erase time (*t*_ERS_) is reduced by up to two orders of magnitude under the same applied bias. These improvements are achieved without compromising compatibility with existing fabrication processes, making the BE filler design a highly practical and scalable solution for future high-density ferroelectric VNAND applications.

Fig. 8(a) and (b) present the *I*–*V* transfer characteristics and the resulting memory window (M. W.) for various filler structures under a fixed erase time condition (*t*_ERS_). This comparison reveals how band-engineered (BE) filler stacks influence both the erase efficiency and the achievable threshold voltage modulation in Fe-VNAND devices. In the case where the P^+^ filler is completely absent, the device fails to perform any meaningful erase operation due to the absence of a vertical conduction path for hole injection. As a result, the cell remains in a neutral state, exhibiting *I*–*V* characteristics nearly identical to those observed in the reference curve shown in Fig. 3(c), where no significant polarization switching occurs. In contrast, as the proportion of nitride (N) material in the oxide-nitride composite filler increases, a noticeable improvement in both channel potential and polarization efficiency is observed. This enhancement directly translates to a wider M. W., with an approximate 30% increase compared to the SiO_2_-only baseline case. The introduction of nitride lowers the energy barrier for hole tunneling, allowing stronger and more uniform polarization switching across the ferroelectric layer. This improved modulation of the channel potential makes it possible to induce deeper erase states and stronger threshold voltage separation, which are essential for enabling multilevel memory operation and improving bit-level sensing accuracy. However, it is important to note that aggressive thinning of the oxide layer, down to approximately 1 nm, while beneficial for tunneling current, also introduces challenges. In this condition, the *V*_th_ shifts excessively, reaching levels that surpass the sensing capability at *V*_read_ = 3 V. This behavior, while indicative of strong polarization, can limit the practical readout unless the read voltage is adjusted accordingly or circuit-level compensation is introduced. Therefore, a trade-off must be considered between maximizing polarization and maintaining read margin, particularly in highly scaled architectures. To further quantify the impact of BE filler engineering, an additional analysis was performed by adjusting the *t*_ERS_ across all filler configurations to align their output *V*_th_ to a common target level. Fig. 8(c) illustrates the point of alignment, and Fig. 8(d) summarizes the corresponding *t*_ERS_ required for each structure to achieve that common *V*_th_. The results clearly demonstrate a remarkable trend: as the nitride fraction increases, the required erase time drops dramatically—from 50 µs in the oxide-only structure, to 23 µs, 11 µs, and ultimately just 0.5 µs in the most optimized BE configuration. This translates to a 100-fold improvement in erase speed, achieved without sacrificing memory window or structural compatibility with conventional TCAT process flows. These results collectively highlight a crucial design insight: band-engineered filler structures can be strategically tuned either to maximize memory window under fixed operation time, or to dramatically reduce erase time while maintaining target threshold modulation. Although the 1/6/1 nm ONO split offered the strongest ERS performance, 2/4/2 nm is recommended as a practical optimum due to realistic process constraints on ultrathin oxide formation. In practical terms, this flexibility enables device designers to optimize for performance (high-speed operation) or reliability (large sensing margin) depending on system-level requirements. Furthermore, since the BE filler concept involves only material and geometric reconfiguration within the existing stack, it is highly manufacturable and scalable for future 3D memory technologies. Thus, BE filler engineering emerges not only as a physical enabler of low-voltage ferroelectric switching, but also as a critical knob for performance-reliability co-optimization in vertical Fe-VNAND architectures.

## Conclusion

In summary, this work presents an a-IGZO channel-based Fe-VNAND structure integrated with a novel erase biasing scheme and band-engineered filler design. By introducing two dummy word-lines at the bottom of the stack, the proposed erase scheme effectively alleviates the over-erased issue at the lower select word-line (SWL_L), thereby restoring BL sensing capability. Additionally, optimization of the doping overlap (*L*_ov_) and read voltage (*V*_read_) enables substantial reduction in read disturbance, improving reliability by a factor of eight. Moreover, the application of band-engineered filler insulators enhances hole injection, achieving up to 30% larger memory window and 100× faster erase speed compared to conventional designs. These improvements were obtained without sacrificing stack compatibility, making the proposed techniques readily adaptable to existing TCAT-based fabrication flows. Overall, the proposed strategies demonstrate strong potential for enabling low-voltage, high-reliability operation in future high-density ferroelectric NAND architectures, offering meaningful implications for advanced non-volatile memory and neuromorphic computing platforms.

## Conflicts of interest

The authors declare that there are no financial or any other types of conflicts of interest to declare for this submission.

## Appendix

**Fig. 9:**
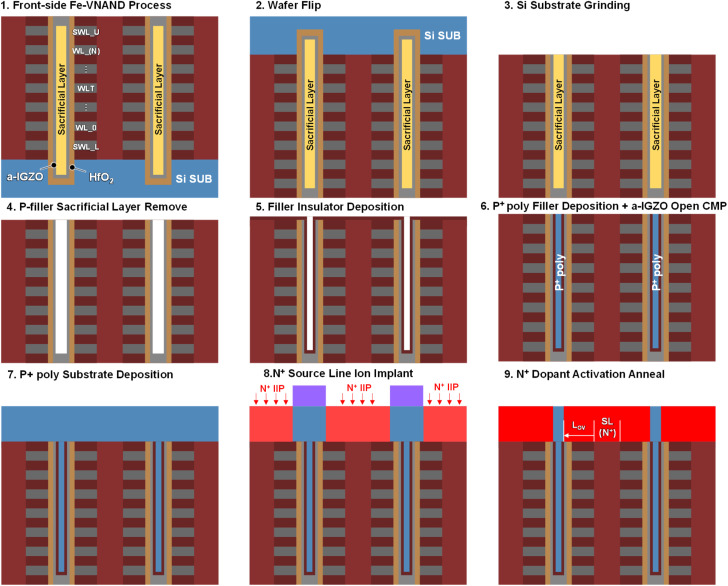
Fabrication sequence of bonding-friendly Fe-VNAND with a-IGZO channel and P^+^ poly-Si filler, comprising (1) BICS-based vertical NAND formation with sacrificial filler oxide, (2) wafer bonding and flip for front-side processing, (3) controlled wafer thinning to expose the filler region, (4) selective removal of sacrificial pillar-support oxide, (5) deposition of oxide or ONO isolation layer—where ONO is employed for band-engineered hole injection during erase, (6) P^+^ poly-Si filling followed by CMP to open the IGZO-poly interface, (7) epitaxial growth of a P^+^ poly-Si substrate, (8) n^+^ source-line implantation, and (9) activation anneal to optimize the overlap length (L_OV) for improved erase and read performance.

Fig. 9 depicts the proposed fabrication flow designed to realize a bonding-friendly a-IGZO channel Fe-VNAND. Step (1) starts with a vertical NAND string fabricated *via* the conventional BICS process, ensuring maximum compatibility with existing high-volume manufacturing lines. This initial step mirrors the baseline approach widely adopted in industry, enabling straightforward integration without major disruption to front-end process infrastructure.

In step (2), the wafer is bonded to a carrier and flipped, a key feature in bonding VNAND integration that allows subsequent process modules to be executed from the front side. This approach is especially advantageous for ultra-high stack counts, as it mitigates aspect-ratio limitations associated with conventional etching from the original substrate side. Step (3) thins the wafer to a controlled depth to access the filler region, ensuring uniform exposure while minimizing damage to surrounding structures. Step (4) selectively removes the sacrificial oxide filler, which had originally served as a mechanical support for the vertical channel pillars during earlier process stages. This removal creates a well-defined cavity for the P^+^ poly-Si filler. Step (5) deposits an oxide or ONO isolation layer between the a-IGZO channel and the P^+^ filler. In the ONO case, the embedded nitride layer enables band engineering by reducing the effective valence band offset, thereby enhancing hole tunneling efficiency during erase and improving polarization reversal in the ferroelectric layer. In step (6), P^+^ poly-Silicon is deposited to fill the cavity, followed by CMP to expose the top of the IGZO-poly interface with high planarity and minimal dishing. Step (7) involves epitaxial growth of the P^+^ poly-Si substrate, establishing a robust vertical conduction path for hole injection. Step (8) patterns and implants the n^+^ source-line region, while step (9) applies an activation anneal to extend the source-line overlap length (L_OV). This deliberate L_OV tuning improves read current drivability at reduced bias conditions, ultimately enabling lower pass/read voltages for effective disturb mitigation. By combining the proven BICS baseline with wafer-flip-enabled front-side processing, this flow not only supports integration of P-filler-based Fe-VNAND in the bonding VNAND era but also enables structural and electrical optimizations—such as enhanced hole injection *via* ONO band engineering and overlap control—that are critical for low-voltage, high-reliability operation in ferroelectric material based next-generation high-density memory stacks.

## Data Availability

The data that support the findings of this study are available from the corresponding author upon reasonable request. All measurement and calibration datasets generated and analysed in this study are available. All research data, including the complete device structures used, are openly provided. The full simulation codes developed for this work are also available.
